# Clinical utility of the MAF-5 score for assessing MAFLD/MASLD in a Japanese population with obesity

**DOI:** 10.1007/s13340-025-00874-2

**Published:** 2026-01-21

**Authors:** Hayato Fukumitsu, Kazuhiko Sakaguchi, Marika Nishisaka, Yukari Katsura, Yasuko Morita, Natsu Otowa-Suematsu, Tomoko Yamada, Yoshihiko Yano, Michiko Takahashi, Shun-Ichiro Asahara, Wataru Ogawa

**Affiliations:** 1 Division of Diabetes and Endocrinology, Department of Internal Medicine, Kobe University Graduate School of Medicine, 7-5-1 Kusunoki-cho, Chuo-ku, Kobe, 650-0017 Japan; 2 Division of Community Medicine and Medical Education, Department of Social/Community Medicine and Health Science, Kobe University Graduate School of Medicine, 7-5-1 Kusunoki-cho, Chuo-ku, Kobe, 650-0017 Japan; 3 Department of Gastroenterology, Konan Medical Center, 1-5-16 Kamokogahara, Higashinada-ku, Kobe, 658-0064 Japan; 4 Department of Nutrition, Kobe University Hospital, 7-5-2 Kusunoki-cho, Chuo-ku, Kobe, 650-0017 Japan; 5 Division of Metabolic Diseases, Department of Translational Medical Science, Kobe University Graduate School of Medicine, 7-5-1 Kusunoki-cho, Chuo-ku, Kobe, 650-0017 Japan

**Keywords:** MAF-5, FIB-4 index, MASLD, VCTE

## Abstract

**Background:**

Metabolic dysfunction-associated steatotic liver disease (MASLD) coexists with type 2 diabetes and is rising in Japan. Liver fibrosis progression in MASLD causes adverse outcomes, highlighting the need for early risk stratification. The utility of the Metabolic Dysfunction–Associated Fibrosis 5 (MAF-5) score has not been assessed in Japanese populations, especially among individuals with type 2 diabetes. Herein, the clinical relevance of the MAF-5 score was assessed in Japanese patients with MASLD.

**Methods:**

This prespecified secondary analysis used data from a study titled “A Study to Estimate the Severity of MAFLD Using Continuous Glucose Monitoring.” Sixty-six patients diagnosed with metabolic dysfunction–associated fatty liver disease (MAFLD) underwent vibration-controlled transient elastography. All participants were subsequently confirmed to meet the revised diagnostic criteria for MASLD. The MAF-5 score and FIB-4 index were calculated for each participant. Correlations between these scores and liver stiffness measurement (LSM) were assessed using Spearman’s rank correlation coefficient. Significant fibrosis was defined as LSM ≥ 8.0 kPa. The predictive performance of each score was evaluated using the area under the receiver operating characteristic curve (AUROC).

**Results:**

The final analysis included 57 participants (28 with type 2 diabetes). The MAF-5 score significantly correlated with LSM, whereas the FIB-4 index did not. These associations were consistent regardless of diabetes status. The AUROC for the MAF-5 score was higher than that for the FIB-4 index.

**Conclusion:**

The MAF-5 score may serve as a useful noninvasive marker for predicting liver fibrosis in Japanese patients with MASLD, regardless of diabetes status.

## Introduction

Metabolic dysfunction-associated steatotic liver disease (MASLD) frequently coexists with type 2 diabetes and is increasingly prevalent worldwide, including in Japan [1]. As liver fibrosis progresses, clinical outcomes worsen, with increased risks of cirrhosis and hepatocellular carcinoma (HCC) [2, 3]. Notably, cardiovascular disease incidence also increases with fibrosis progression [4, 5]. Therefore, early identification of high-risk individuals is a critical clinical objective.

Histological diagnosis via liver biopsy remains the gold standard for liver fibrosis assessment. However, this approach is invasive and subject to sampling variability [6]. Vibration-controlled transient elastography (VCTE) is a non-invasive alternative for quantifying liver fibrosis through liver stiffness measurement (LSM) and hepatic steatosis via the controlled attenuation parameter (CAP), with proven diagnostic accuracy [7]. However, its requirement for specialized equipment limits use in primary care and general health-screening settings. Hence, a critical need exist for accessible screening tools that can identify individuals at high risk of advanced fibrosis.

The Fibrosis-4 index (FIB-4 index), calculated using age, platelet count, aspartate aminotransferase (AST) and alanine aminotransferase (ALT) levels, correlates with liver-related mortality, cirrhosis progression, and HCC development [8–10]. Given its low cost, repeatability, and usefulness in monitoring longitudinal changes and therapeutic responses, FIB-4 index is widely recommended for initial screening in clinical practice guidelines.

However, the diagnostic performance of the FIB-4 index is diminished in older adults, individuals with obesity, and patients with type 2 diabetes when compared with histological findings [11–13]. These limitations have prompted efforts to develop and validate more accurate non-invasive tests (NITs) for fibrosis assessment in populations with metabolic dysfunction.

Metabolic Dysfunction–Associated Fibrosis 5 (MAF-5) score, incorporating AST level, platelet count, body mass index (BMI), diabetes status, and waist circumference, is a newly proposed tool [14]. Originally developed in Western populations, MAF-5 score strongly correlates with histological fibrosis, VCTE-based assessments, and liver-related outcomes such as hospitalization, HCC incidence, and mortality [14–16].

To date, however, no studies have evaluated the utility of MAF-5 score in Japanese populations, particularly among individuals with type 2 diabetes. Therefore, this study assessed the clinical relevance of the MAF-5 score in Japanese patients with MASLD by examining its association with CAP and LSM values from VCTE (FibroScan®), and comparing its diagnostic performance with other NITs.

## Material and methods

### Study design

This prespecified secondary analysis is based on “A Study to Estimate the Severity of MAFLD Using Continuous Glucose Monitoring.” The study was conducted in accordance with the Declaration of Helsinki, approved by the Ethics Committee of Kobe University Hospital (Approval No. B230230), and registered with UMIN (UMIN000053360).

### Study participants

Study participants were patients who had provided written informed consent to participate in a randomized, double-blind, placebo-controlled trial titled “A Phase III, Randomized, Double-Blind, Placebo-Controlled Trial to Investigate the Effect of a Brown Rice-Derived Supplement on Hepatic Steatosis in Patients with Metabolic Dysfunction-Associated Fatty Liver Disease (MAFLD).” This study was registered in the Japan Registry of Clinical Trials (registration number: jRCTs051230187). Inclusion criteria were as follows: (1) MAFLD diagnosis according to established criteria [17]; (2) age ≥ 18 years at the time of consent; and (3) a controlled attenuation parameter (CAP) ≥ 230 dB/m as assessed by VCTE. Exclusion criteria were as follows: (1) history of cancer treatment within the past 5 years; (2) known allergy to rice or starch; (3) current pregnancy; (4) presence of implantable medical devices; and (5) any other condition deemed inappropriate for study participation by the principal investigator or sub-investigators. Written informed consent was obtained from all study participants. Although study participants were initially enrolled based on the diagnostic criteria for metabolic dysfunction–associated fatty liver disease (MAFLD), all individuals were subsequently confirmed to meet the recently proposed criteria for metabolic dysfunction–associated steatotic liver disease (MASLD) [18]. Therefore, the term MASLD is used throughout this manuscript.

### Assessments and outcomes

Eligible participants who met all inclusion criteria underwent blood testing, anthropometric measurements, and liver fibrosis assessment using VCTE (FibroScan®, Echosens, Paris, France). The M or XL probe was selected according to body weight (XL for ≥ 100 kg) and/or as recommended by the device's automated probe selection tool. Liver fibrosis was defined as an LSM ≥ 8.0 kPa, as recommended by the international guideline [19]. Only VCTE data with an interquartile range-to-median ratio (IQR/med) < 30% were included in the analysis.

Each NIT was calculated using the following formulas:

- MAF-5 score:

-11.3674 + (waist circumference [WC, cm] × 0.0282) − (BMI [kg/m^2^] × 0.1761) + (WC × BMI × 0.0019) + 2.0762 (if diabetes present) + ln[AST (U/L)] × 2.9207 − platelet count (10^9^/L) × 0.0059 [14]

- FIB-4 index:

(age × AST [U/L])/(platelet count [10^9^/L] × √ALT [U/L]) [8]

- FIB-3 index:

5 × ln[AST (U/L)] − 2 × ln[ALT (U/L)] − 0.18 × platelet count (10^4^/μL) − 5 [20]

- APRI (AST to Platelet Ratio Index):

(AST [U/L]/upper limit of normal AST)/platelet count [10^9^/L] × 100 [21]

### Statistical analyses

Statistical analyses were performed using IBM SPSS Statistics for Windows (version 30.0, IBM Corp., Armonk, NY, USA), GraphPad Prism (version 10.4.2; GraphPad Software, San Diego, CA, USA), and R (version 4.3.1; R Foundation for Statistical Computing, Vienna, Austria). Categorical variables were compared between groups using the chi-squared test or Fisher’s exact test, as appropriate. The Shapiro–Wilk test assessed the normality of data distribution. Continuous variables between two independent groups were compared using the unpaired *t*-test for normally distributed data, and the Mann–Whitney *U* test was applied for non-normally distributed data. Pearson correlation was used for normally distributed data in correlation analyses, and Spearman rank correlation was applied for non-normally distributed data. These analyses were conducted to evaluate the associations between VCTE results and each NIT, including MAF-5 score, FIB-4 index, FIB-3 index, and APRI. The diagnostic performance of the MAF-5 score for detecting liver fibrosis was assessed using receiver operating characteristic (ROC) curve analysis and compared with that of the FIB-4 index based on the area under the curve (AUC) with DeLong’s test. A two-tailed *p*-value < 0.05 was considered statistically significant.

## Results

VCTE was performed in 66 patients with MAFLD, and 57 who met the criteria of a CAP ≥ 230 dB/m and an IQR/med < 30% were included in the analysis.

The baseline characteristics of study participants are summarized in Table 1. The mean age of participants was 50.7 ± 11.9 years, mean body mass index (BMI) was 34.2 ± 6.5 kg/m^2^, and mean HbA1c was 6.3 ± 0.7%. The mean MAF-5 score, FIB-4 index, FIB-3 index, and APRI were 2.1 ± 2.6, 1.0 ± 0.5, − 0.3 ± 1.8, and 0.4 ± 0.3, respectively. The mean LSM was 6.9 ± 3.7 kPa, and 10 out of 57 patients (17.5%) were classified as having liver fibrosis, defined as an LSM ≥ 8.0 kPa. No significant differences were observed between the diabetes (*n* = 29) and non-diabetes (*n* = 28) groups regarding age, sex, body weight, BMI, CAP, or LSM. The platelet count was lower in the non-diabetes group than that in the diabetes group (249.2 ± 46.0 vs. 272.6 ± 50.9 × 10^9^/L), although the difference was not significant (*p* = 0.075).

**Table 1:** Clinical characteristics of study participants

	All (n = 57)	Diabetes (n = 29)	Non-diabetes (n = 28)	*p*-value
Age (years)	50.7 ± 11.9	52.9 ± 12.3	48.4 ± 11.4	0.160
Male, n (%)	20 (35.1)	12 (41.4)	8 (28.6)	0.311
Weight (kg)	90.5 ± 20.8	89.9 ± 21.5	91.1 ± 20.5	0.825
BMI (kg/m^2^)	34.2 ± 6.5	33.9 ± 7.2	34.5 ± 5.8	0.587
Waist circumference (cm)	110.8 ± 15.2	110.6 ± 16.5	110.9 ± 14.0	0.917
Non**-**significant alcohol consumption^a^, n (%)	24 (42.1)	17 (58.6)	7 (25.0)	0.010
Complications				
Hypertension, n (%)	25 (43.9)	15 (51.7)	10 (35.7)	0.223
Hyperlipidemia, n (%)	47 (82.5)	25 (86.2)	22 (78.6)	0.504
Hyperuricemia, n (%)	11 (19.3)	3 (10.3)	8 (28.6)	0.081
Sleep apnea syndrome, n (%)	13 (22.8)	6 (20.7)	7 (25.0)	0.698
Musculoskeletal disorders, n (%)	13 (22.8)	6 (20.7)	7 (25.0)	0.698
Laboratory data				
PLT (10^9^/L)	261.1 ± 49.5	272.6 ± 50.9	249.2 ± 46.0	0.075
AST (U/L)	29.2 ± 17.9	27.0 ± 16.7	31.5 ± 19.1	0.098
ALT (U/L)	40.7 ± 37.0	38.5 ± 34.6	42.9 ± 39.7	0.615
γ**-**GTP (U/L)	52.1 ± 85.7	61.3 ± 113.5	42.5 ± 41.1	0.492
FPG (mg/dL)	115.6 ± 34.1	132.2 ± 40.2	98.3 ± 11.5	< 0.001
HbA1c (%)	6.3 ± 0.7	6.7 ± 0.8	5.8 ± 0.4	< 0.001
TG (mg/dL)	152.9 ± 108.7	154.0 ± 130.7	151.9 ± 82.5	0.604
LDL**-**C (mg/dL)	115.7 ± 27.4	109.0 ± 30.0	122.6 ± 22.9	0.059
HDL**-**C (mg/dL)	57.5 ± 14.4	58.2 ± 16.1	56.8 ± 12.6	0.987
Total cholesterol (mg/dL)	194.4 ± 29.5	187.2 ± 31.2	201.9 ± 26.1	0.058
UA (mg/dL)	5.7 ± 1.2	5.3 ± 1.1	6.1 ± 1.1	0.007
Creatinine (mg/dL)	0.7 ± 0.2	0.8 ± 0.3	0.7 ± 0.2	0.661
eGFR (mL/min/1.73m^2^)	79.3 ± 23.9	78.8 ± 26.0	79.9 ± 21.9	0.817
Non-invasive tests				
MAF-5 score	2.1 ± 2.6	2.8 ± 2.4	1.3 ± 2.6	0.008
FIB-4 index	1.0 ± 0.5	0.9 ± 0.4	1.0 ± 0.5	0.534
FIB-3 index	− 0.3 ± 1.8	− 0.8 ± 1.7	0.2 ± 1.6	0.019
APRI	0.4 ± 0.3	0.4 ± 0.3	0.4 ± 0.2	0.039
Vibration-controlled transient elastography				
M Probe, n (%)	42 (73.7)	21 (72.4)	21 (75.0)	0.825
CAP (dB/m)	306.2 ± 48.9	302.7 ± 47.1	309.9 ± 51.4	0.587
LSM (kPa)	6.9 ± 3.7	7.4 ± 4.1	6.3 ± 3.3	0.411

^a^Non-significant alcohol consumption was defined as < 210 g/week of pure alcohol for men and < 140 g/week for women

*BMI* body mass index, *PLT* platelet count, *AST* aspartate aminotransferase, *ALT* alanine aminotransferase, *γ-GTP* gamma-glutamyl transpeptidase, *FPG* fasting plasma glucose, *HbA1c* hemoglobin A1c, *TG* triglyceride, *LDL-C* low-density lipoprotein cholesterol, *HDL-C* high-density lipoprotein cholesterol, *UA* uric acid, *eGFR* estimated glomerular filtration rate, *MAF-5* metabolic dysfunction-associated fibrosis, *FIB-4* fibrosis-4, *FIB-3* fibrosis-3, *APRI* AST to platelet ratio index, *CAP* controlled attenuation parameter, *LSM* liver stiffness measurement

Continuous variables are presented as the mean ± standard deviation and compared between groups using the Student’s *t*-test or Mann–Whitney *U* test, as appropriate. Categorical variables are presented as numbers (%) and compared using the chi-squared test or Fisher’s exact test. Non-significant alcohol consumption was defined as < 210 g/week of pure alcohol for men and < 140 g/week for women

**Fig. 1:**
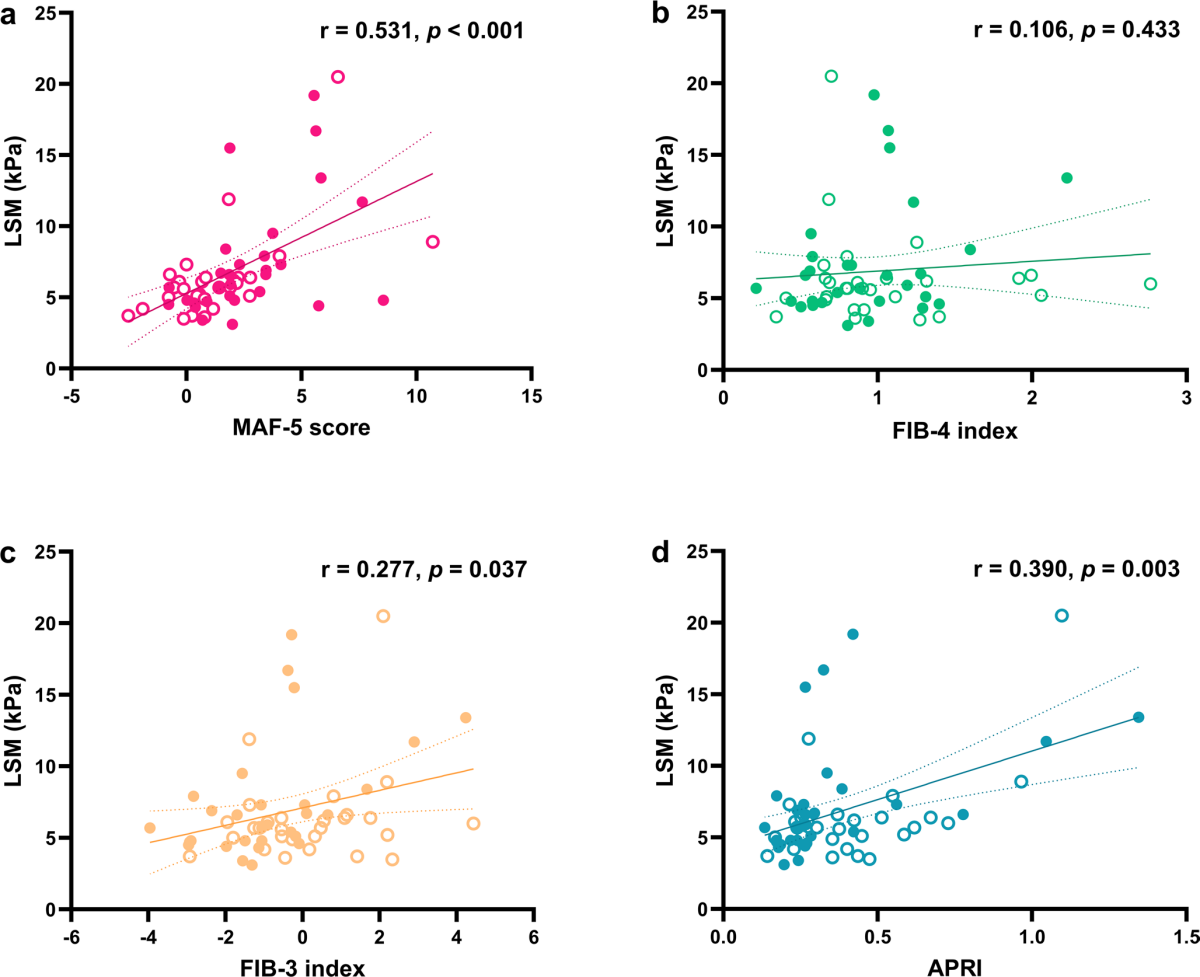
Correlation between liver stiffness measurement (LSM) and: **a** MAF-5 score, **b** FIB-4 index, **c** FIB-3 index, and **d** APRI. Each data point represents an individual participant. Filled circles indicate patients with diabetes; open circles indicate patients without diabetes. The solid line represents linear regression; dotted lines indicate the 95% confidence interval. Spearman’s correlation coefficient (r) and p-value are shown

A significant positive correlation was observed between LSM and the MAF-5 score (*r* = 0.531, *p* < 0.001), whereas no significant correlation was found between LSM and the FIB-4 index (*r* = 0.106, *p* = 0.433) (Fig. 1a, b). The FIB-3 index (*r* = 0.277, *p* = 0.037) and APRI (*r* = 0.390, *p* = 0.003) also showed significant correlations with LSM (Fig. 1c, d); however, among the four non-invasive fibrosis scores, the MAF-5 score demonstrated the strongest correlation.

Subgroup analyses were performed based on diabetes status (Table 2). Among patients with diabetes (*n* = 29), LSM remained significantly correlated with the MAF-5 score (*r* = 0.470, *p* = 0.010), FIB-3 index (*r* = 0.454, *p* = 0.013), and APRI (*r* = 0.581, *p* = 0.001); however, it did not correlate with the FIB-4 index (*r* = 0.212, *p* = 0.269). In patients without diabetes (*n* = 28), LSM was significantly associated only with the MAF-5 score (*r* = 0.529, *p* = 0.004). No significant correlations were observed between LSM and the FIB-4 index (*r* = –0.008, *p* = 0.969), FIB-3 index (*r* = 0.111, *p* = 0.574), or APRI (*r* = 0.272, *p* = 0.162). For CAP, only the MAF-5 score consistently showed a significant positive correlation across all subgroups.

**Table 2:** Correlation coefficients (*r*) between non-invasive tests (NITs) and vibration-controlled transient elastography (VCTE) parameters

NITs	VCTE parameter	All	Diabetes	Non-Diabetes
		(n = 57)	(n = 29)	(n = 28)
MAF-5 score	CAP	0.423**	0.429*	0.453*
	LSM	0.531***	0.470*	0.529**
FIB-4 index	CAP	− 0.118	0.001	− 0.255
	LSM	0.106	0.212	− 0.008
FIB-3 index	CAP	0.104	0.274	− 0.109
	LSM	0.277*	0.454*	0.111
APRI	CAP	0.257	0.389*	0.157
	LSM	0.390**	0.581**	0.272

*NITs* non-invasive tests, *VCTE* vibration-controlled transient elastography, *MAF-5* metabolic dysfunction-associated fibrosis, *FIB-4* fibrosis-4, *FIB-3* fibrosis-3, *APRI* AST to platelet ratio index, *CAP* controlled attenuation parameter, *LSM* liver stiffness measurement

Data represent Spearman’s rank correlation coefficients. *p* < 0.05: *; *p* < 0.01: **; *p* < 0.001: ***

**Fig. 2:**
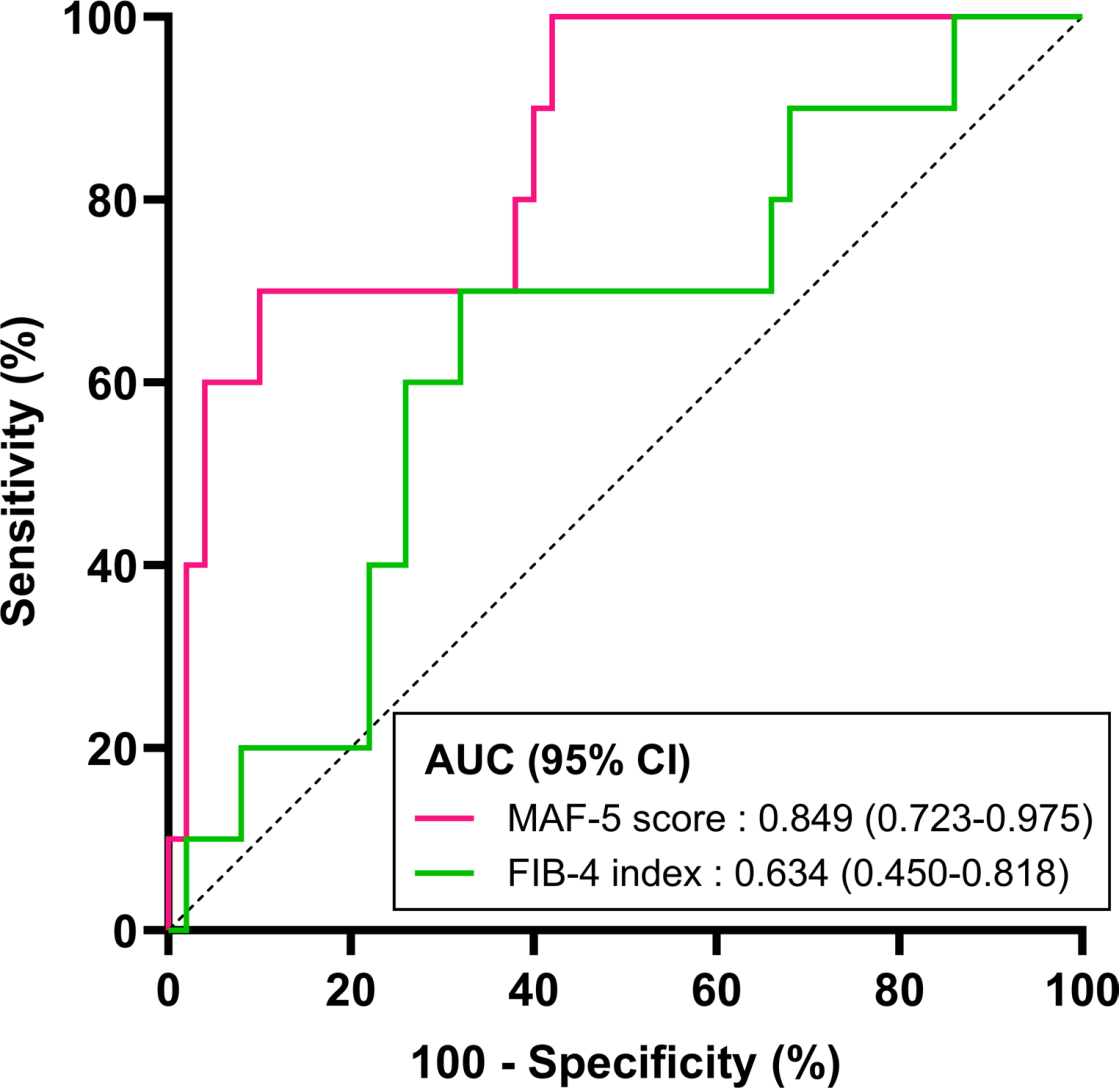
Receiver operator curve analysis comparing the ability of MAF-5 score and FIB-4 index to identify patients with a liver stiffness measurement ≥ 8.0 kPa

Finally, ROC curve analysis for predicting significant fibrosis demonstrated that the AUC for the MAF-5 score was higher than that for the FIB-4 index, although the difference did not reach statistical significance (0.849 (95% CI 0.723–0.975) vs. 0.634 (95% CI 0.450–0.818), *p* = 0.065) (Fig. 2). The optimal cutoff value for the MAF-5 score, determined by maximizing the Youden index, was 3.6, corresponding to a sensitivity of 70.0% and a specificity of 89.4%. This threshold provides a balanced diagnostic performance and can be regarded as a rule-in-oriented cutoff for identifying individuals at high likelihood of liver fibrosis. However, because a single cutoff optimized for the Youden index does not necessarily reflect different clinical decision-making contexts, such as screening versus confirmatory assessment, we additionally adopted a dual-cutoff strategy to enhance clinical interpretability. In particular, for screening purposes, minimizing false-negative results is often prioritized over overall diagnostic accuracy. Accordingly, we examined a lower cutoff value emphasizing sensitivity. A cutoff of 1.6 achieved 100% sensitivity while maintaining a comparatively high Youden index (0.574) and a specificity of 57.4%. This lower threshold may therefore be more suitable for rule-out applications, allowing reliable exclusion of advanced fibrosis at the screening stage, whereas the higher cutoff may be reserved for confirmatory decision-making, together constituting a clinically pragmatic dual-cutoff strategy.

## Discussion

This study demonstrated that the MAF-5 score, originally derived in Western populations, closely correlates with LSM in Japanese patients with MASLD regardless of diabetes status and may outperform the FIB-4 index in predicting hepatic fibrosis [12]. Although the MAF-5 score includes variables such as BMI and waist circumference, which vary across ethnicities, it remained a useful and robust fibrosis indicator in an Asian population.

To our knowledge, this report is the first to indicate that MAF-5 score could serve as a more accurate non-invasive liver fibrosis marker than the FIB-4 index in a Japanese MASLD cohort including individuals with diabetes and obesity.

In this study, liver stiffness was assessed using VCTE, a widely adopted noninvasive diagnostic modality for evaluating hepatic fat content and stiffness. VCTE is painless, repeatable, and well-tolerated, and its derived parameters predict liver-related outcomes in patients with MAFLD [7, 22].

Despite its clinical utility, VCTE is a high-cost device with limited availability, restricting its routine use in general practice. Consequently, a growing need exists for simple, accessible surrogate markers that can reliably reflect VCTE results and serve as first-line screening tools for hepatic fibrosis in broader clinical settings.

The FIB-4 index, commonly used as a first-line liver fibrosis screening tool in MASLD [19, 23], exhibits reduced predictive accuracy in patients with diabetes [11–13]. Diabetes is an independent risk factor for fibrosis progression in patients with MASLD [24, 25]. Given that the MAF-5 score includes diabetes status as one of its variables, it may offer superior diagnostic performance than the FIB-4 index, particularly in populations that include individuals with diabetes.

The MAF-5 score may predict liver-related events even in the general population [15]. As a simple, noninvasive tool derived from routine clinical parameters, the MAF-5 score may more efficiently identify high-risk individuals than the FIB-4 index, even in Japanese populations. Furthermore, a recent study involving a large multiethnic MAFLD cohort showed that the MAF-5 score accurately detected liver stiffness ≥ 8.0 kPa on VCTE, with an area under the receiver operating curve (AUROC) of 0.81, consistent with our findings [14]. In contrast, a prior study targeting a high-risk MAFLD population—predominantly composed of individuals with type 2 diabetes or obesity—reported a high false-positive rate with the MAF-5 score, raising concerns about its positive predictive value in such subgroups [26]. As the MAF-5 score was originally developed for general population screening, further validation is necessary to determine its clinical utility in high-risk cohorts.

Although MAF-5 demonstrated the strongest correlation with LSM, both APRI and FIB-3 were significantly associated with LSM in the overall cohort, and APRI showed particularly strong correlations in patients with diabetes. These findings highlight that multiple NITs may capture different aspects of fibrosis biology and may be useful in specific clinical contexts. However, the differences in statistical significance across subgroups may also reflect the limited sample size. Nevertheless, MAF-5 was the only index showing robust correlations across both diabetes and non-diabetes subgroups, suggesting broader applicability in heterogeneous MASLD populations. Our study demonstrated the MAF-5 score may still be useful in patients with type 2 diabetes; however, the generalizability of these findings should be interpreted with caution, and larger prospective studies are warranted.

This study had some limitations. First, the findings were derived from exploratory analysis of secondary data rather than a pre-specified primary endpoint, warranting results interpretation as hypothesis-generating and requiring further validation in prospective confirmatory studies. Nevertheless, such exploratory analyses are valuable in identifying potential associations and guiding future research design. Second, the study population was skewed toward patients with relatively mild MAFLD, and most participants exhibited low FIB-4 index values, potentially underestimating the diagnostic performance of the FIB-4. Third, because the MAF-5 score incorporates both BMI and waist circumference as core components of its algorithm, its sensitivity may be reduced in individuals with lean MASLD, who may exhibit considerable hepatic fibrosis despite having normal anthropometric parameters. Moreover, the limited number of cases with LSM ≥ 8.0 kPa may have led to overestimation of AUC values in ROC analyses, highlighting the need for caution in generalizing the diagnostic accuracy of these findings. To more rigorously assess the clinical utility of the MAF-5 score in comparison with other noninvasive markers, larger studies encompassing more diverse populations and a wider range of fibrosis severity are warranted.

In conclusion, these findings suggest that the MAF-5 score may complement or serve as an alternative to existing fibrosis screening tools, particularly in settings with limited access to VCTE. Its simplicity and broad applicability highlight its potential as a first-line assessment tool in routine clinical care.

## Data Availability

The datasets used and analyzed during this study are available from the corresponding author upon request.
